# Disruption of Histidine Biosynthesis Impairs Outer Membrane Stability and Intracellular Survival of *Brucella melitensis*, Resulting in Attenuated Virulence

**DOI:** 10.3390/microorganisms14061323

**Published:** 2026-06-12

**Authors:** Yang Li, Qiumei Shi, Guangyu Yang, Simin Chen, Jinyue Liu, Na Li, Li Chen, Zhenhua Wang, Run Li, Jiao Wang, Shaohui Wang, Yanqing Bao, Jingjing Qi, Tonglei Wu, Mingxing Tian

**Affiliations:** 1 Shanghai Veterinary Research Institute, Chinese Academy of Agricultural Sciences, Shanghai 200241, China; 15255936477@163.com (Y.L.); yanggy3580@163.com (G.Y.); csmin111@163.com (S.C.); liujinyue0630@163.com (J.L.); ln2001520@163.com (N.L.); shwang@shvri.ac.cn (S.W.); ybao@shvri.ac.cn (Y.B.); qijingjing@shvri.ac.cn (J.Q.); 2 Hebei Key Laboratory of Preventive Veterinary Medicine, College of Animal Science and Technology, Hebei Normal University of Science and Technology, Qinhuangdao 066600, China; shiqiumei@126.com (Q.S.); 18310985287@163.com (L.C.); 3 Shijiazhuang Shengbo Biological Technology Co., Ltd., Shijiazhuang 050031, China; 308363691@qq.com; 4 Tangshan Yian Biological Engineering Co., Ltd., Tangshan 063000, China; lr@yian-bio.cn; 5 Beijing Nabai Biotechnology Co., Ltd., Beijing 100176, China; 411515882@qq.com

**Keywords:** *Brucella melitensis*, *hisD*, histidine biosynthesis, virulence, outer membrane stability, intracellular survival

## Abstract

**Background:** Brucellosis is a global zoonosis caused by *Brucella*. Histidine biosynthesis is essential for bacterial growth, but its role in *Brucella melitensis* virulence remains unclear. HisD catalyzes the final two steps of histidine synthesis and is absent in mammals, making it a potential drug target. **Results:** We constructed a *hisD* deletion mutant (Δ*hisD*) and complemented strain (C*hisD*) via homologous recombination. Δ*hisD* failed to grow in medium without histidine supplementation. It showed reduced survival under polymyxin B and SDS stress, and impaired outer membrane integrity under polymyxin B challenge, though no defect was observed under non-stressed conditions. Intracellularly, Δ*hisD* replicated poorly in HeLa and RAW264.7 cells, and this defect was rescued by exogenous histidine. In a mouse model, Δ*hisD* exhibited lower bacterial loads in liver and spleen, reduced splenomegaly, and attenuated hepatic granuloma formation. **Conclusions:** Histidine biosynthesis deficiency attenuates *Brucella* virulence by restricting nutritional acquisition and conditionally compromising outer membrane stability. HisD is a promising target for anti-brucellosis drug development, and Δ*hisD* holds potential as a live attenuated vaccine candidate.

## 1. Introduction

Brucellosis is an important bacterial zoonosis caused by infection with *Brucella*. Infected animals typically exhibit abortion in pregnant females, stillbirth, and orchitis in males. In humans, infection is characterized by influenza-like symptoms, including undulant fever, sweating, and fatigue; chronic cases may lead to various complications such as arthritis, orchitis, hepatitis, encephalomyelitis, spondylitis, and splenomegaly [1]. *Brucella* is a Gram-negative coccobacillus and a facultative intracellular pathogen capable of surviving inside multiple host cell types, including macrophages, dendritic cells, and trophoblasts [2,3]. Following infection, *Brucella* persists within host cells by evading immune recognition and interfering with antigen presentation, thereby establishing chronic infection [4]. Therefore, identifying and functionally characterizing virulence-related factors is essential for understanding the pathogenesis of this pathogen.

Although *Brucella* lacks classical virulence factors such as exotoxins or cytolysins, its virulence-associated components—including lipopolysaccharide (LPS), the type IV secretion system (T4SS), and the two-component regulatory system BvrS/R—play critical roles in resisting host immune killing, promoting intracellular survival, and maintaining membrane homeostasis [2,3,4]. Advances in research technologies have led to the discovery of an increasing number of key genes that directly or indirectly affect *Brucella* virulence. Among them, several genes involved in bacterial metabolism contribute significantly to membrane homeostasis, intracellular survival, nutrient acquisition, and induction of inflammatory responses. For example, deletion of *purD*, which encodes phosphoribosylamine-glycine ligase, disrupts the *de novo* purine nucleotide biosynthesis pathway and thereby affects membrane homeostasis, intracellular survival, and virulence of *B. melitensis* [5]; deletion of the glucose transporter gene *gluP* impairs glucose uptake and the growth of *B. abortus* in the extracellular space of mouse placental cells [6]; and the VdtR-regulated erythronate utilization plays an important role in *Brucella* virulence and induction of placental inflammation in mice [7]. The link between bacterial metabolism and virulence has been recognized for over a decade [8], and collectively, these studies indicate that investigating metabolism-related genes offers a valuable perspective for understanding the pathogenic mechanisms of *Brucella*.

Histidine, an essential amino acid, is not only required for protein synthesis and the acquisition of carbon, nitrogen, and energy, but also participates in two-component signal systems that regulate environmental stress responses and virulence in bacteria [9,10,11]. Bacteria can obtain histidine either from exogenous sources or through *de novo* synthesis, depending on environmental conditions; when histidine is scarce, bacteria initiate the *de novo* biosynthetic pathway. *Brucella* possesses a complete histidine *de novo* biosynthesis pathway, which starts from the precursor 5-phosphoribosyl-1-pyrophosphate (PRPP) and generates L-histidine via ten enzymatic steps (Figure S1). This pathway is highly conserved in *Brucella*. Previous transposon mutant library screens have revealed that deletion of multiple histidine biosynthesis genes, such as those encoding phosphoribosyl-ATP pyrophosphatase (HisE) and histidinol dehydrogenase (HisD), impairs the intracellular survival of *Brucella* [12,13]. HisD catalyzes the ninth and tenth steps of histidine biosynthesis, in which L-histidinol is successively dehydrogenated to L-histidine (Figure S1). It has been reported that HisD represents a candidate target for developing anti-brucellosis drugs, as analogues of its substrate histidinol effectively inhibit the intracellular survival of *B. suis* [14,15]. Antibiotics are critical for treating brucellosis, yet antimicrobial resistance (AMR) is increasingly reported among different *Brucella* species, including *B. melitensis* and *B. abortus*, with high resistance rates to frontline agents such as rifampin and co-trimoxazole [16]. The facultative intracellular lifestyle of *Brucella* further limits the efficacy of many conventional antibiotics [17], highlighting a continued need for novel drugs that target essential bacterial pathways inside host cells. Nevertheless, a systematic investigation of the role of the histidine biosynthesis pathway in *Brucella* virulence is still lacking, which limits the development of pathway-based targeted therapeutics and novel vaccines.

To address this gap, we constructed a histidine-auxotrophic strain of *B. melitensis* by deleting the *hisD* gene. Using assays of bacterial growth curves, tolerance to bactericidal factors, membrane permeability, cell infection, and mouse infection, we systematically evaluated the impact of histidine auxotrophy on virulence-related phenotypes of *Brucella*. This study aims to elucidate the contribution of impaired histidine biosynthesis to the pathogenicity of *B. melitensis*, and the findings will provide a theoretical basis for developing live attenuated vaccines and drug targets based on this metabolic pathway.

## 2. Materials and Methods

### 2.1. Bacterial Strains and Culture Conditions

The attenuated vaccine strain *B. melitensis* M5 and its derivative strains (CVCC, Beijing, China) were cultured in tryptic soy broth (TSB) (BD-Pharmingen, Franklin Lakes, NJ, USA), brucella broth (BB) (Huankai Microbiol., Guangzhou, China), or chemically defined Plommet’s erythritol (PE) medium at 37 °C with 5% CO_2_. The PE medium consisted of the following components: 2.3 g/L K_2_HPO_4_, 3 g/L KH_2_PO_4_, 0.1 g/L Na_2_S_2_O_3_·5H_2_O_2_, 5 g/L NaCl, 0.2 g/L nicotinic acid, 0.2 g/L thiamine, 0.07 g/L pantothenic acid, 0.5 g/L (NH_4_)_2_SO_4_, 0.01 g/L MgSO_4_, 0.1 mg/L MnSO_4_, 0.1 mg/L FeSO_4_, 0.1 mg/L biotin, 1 mM methionine, and 2 g/L erythritol [5]. All manipulations involving attenuated *Brucella* strains were performed in a Biosafety Level 2 (BSL-2) facility at the Shanghai Veterinary Research Institute, Chinese Academy of Agricultural Sciences. *Escherichia coli* DH5α (TIANGEN Biotech, Beijing, China) was cultured in Luria–Bertani (LB) medium at 37 °C. For solid media, 1.5% (*w*/*v*) agar powder (Sangon Biotech, Shanghai, China) was added to the corresponding liquid medium. When required, antibiotics were supplemented at 100 μg/mL ampicillin or 50 μg/mL kanamycin. The strains and plasmids used in this study are listed in Table 1.

### 2.2. Plasmid Construction

The suicide plasmid was constructed as previously described [5]. Briefly, using the M5 genome as a template, the upstream homology arm of *hisD* was amplified by PCR with primers HisD-UF/UR, and the downstream homology arm was amplified with primers HisD-DF/DR. The purified upstream and downstream fragments were then fused by overlap PCR using HisD-UF/DR as primers. The fused fragment was ligated into XbaI-linearized pKB plasmid using a homologous recombination kit (TIANGEN) to generate the suicide plasmid pKB-Δ*hisD*.

The complemented plasmid was constructed using a similar approach [5]. The *hisD* gene fragment, including the complete ORF together with the predicted promoter and terminator regions, was amplified from the M5 genome with primers ChisD-F/R. After gel purification, the fragment was ligated into KpnI- and BamHI-linearized pMiniTn7TK plasmid using a homologous recombination kit, yielding the complemented plasmid pMiniTn7TK-C*hisD*. The primers used above are listed in Table 2.

### 2.3. Construction of the Deletion and Complemented Strains

The *hisD* deletion mutant was constructed as previously reported [5]. Briefly, *B. melitensis* M5 was cultured to logarithmic phase (optical density at 600 nm [OD_600_] = 0.4–0.6). The cells were washed twice with sterile distilled water, resuspended in 10% (*v*/*v*) glycerol water, and used as electrocompetent cells. An aliquot of 1–2 μg of pKB-Δ*hisD* plasmid was added to 100 μL of competent cells, mixed gently, and incubated on ice for 15 min. The mixture was transferred to a pre-chilled electroporation cuvette and electroporated at 2.4 kV and 400 Ω. After electroporation, 1 mL of TSB pre-warmed to 37 °C was added, and the cells were incubated at 37 °C with shaking for 8 h. The culture was then spread onto tryptic soy agar (TSA) plates containing kanamycin for primary selection. Kanamycin-resistant single colonies were propagated in TSB and subsequently plated onto TSA containing 5% (*w*/*v*) sucrose for counter-selection. Single colonies were picked, propagated, and identified by PCR. The resulting *hisD* deletion mutant was designated Δ*hisD*.

The complemented strain was constructed based on miniTn7 transposon-mediated insertion into the intergenic region between *glmS* and *recG* on the *Brucella* chromosome II [18]. Briefly, Δ*hisD* was cultured in TSB to logarithmic phase, and electrocompetent cells were prepared as described above. A mixture of 100 ng pMiniTn7TK-C*hisD* and 100 ng of the helper plasmid pHelp1 was electroporated into Δ*hisD* cells. Transformants were selected on TSA plates containing kanamycin. Single colonies were picked and verified by PCR, and the complemented strain was named C*hisD*.

### 2.4. Growth Curve Determination

*B. melitensis* M5 and its derivative strains were cultured in TSB to logarithmic phase, washed twice with PBS, and adjusted to an OD_600_ of 1.0. The bacterial suspension was inoculated at a 1:10 ratio into 5 mL of TSB, BB, PE, or PE supplemented with different concentrations of histidine, and incubated at 37 °C with shaking at 220 rpm. Starting from the time of inoculation, the OD_600_ was measured every 6 h for a total of 60 h. Growth curves were plotted based on the measured OD_600_ values.

### 2.5. Sensitivity to Stress Factors

To evaluate the effect of *hisD* deletion on the ability of *Brucella* to withstand various stressors, we assessed bacterial tolerance to hydrogen peroxide, sodium nitroprusside (SNP), acidic environment, polymyxin B, and sodium dodecyl sulfate (SDS). The assays were performed as previously described [5]. Briefly, *Brucella* strains were cultured in TSB to logarithmic phase, adjusted to an OD_600_ of 1.0 (approximately 5 × 10^9^ CFU/mL), and diluted to appropriate concentrations with PBS for subsequent experiments.

Hydrogen peroxide and polymyxin B sensitivity: Bacterial suspensions were diluted to 5 × 10^5^ CFU/mL and mixed with an equal volume of different concentrations of hydrogen peroxide (final concentrations 1, 2, and 4 mM) or polymyxin B (final concentrations 0.5, 1, and 2 mg/mL). An equal volume of PBS was used as a control. The mixtures were incubated at 37 °C with shaking for 1 h, serially diluted ten-fold in PBS, spread onto TSA plates, and incubated for 3–5 days. CFUs were counted, and the survival rate was calculated as: survival rate (%) = (CFU in treated group/CFU in PBS control) × 100.

Acid tolerance: Bacterial suspensions were diluted to 2.5 × 10^4^ CFU/mL. A 50-μL aliquot was added to 950 μL of 0.1% peptone water at different pH values (4.5, 5.5, 6.5, and 7.2), with pH 7.2 serving as the control. After incubation at 37 °C with shaking for 2 h, the samples were diluted ten-fold in PBS, spread onto TSA plates, and CFUs were counted. The survival rate was calculated as: survival rate (%) = (CFU in treated group/CFU in control group) × 100.

SDS sensitivity: A 100-μL aliquot of bacterial suspension at an OD_600_ of 1.0 was spread evenly onto TSA or brucella agar (BA) plates. A sterile paper disk was placed in the center of each plate, and 7 μL of 20% (*w*/*v*) SDS solution was added dropwise. The plates were incubated at 37 °C with 5% CO_2_ for 3–5 days, after which the diameter of the inhibition zone was measured.

SNP sensitivity: Bacterial suspensions at an OD_600_ of 1.0 were serially diluted ten-fold. A 2-μL aliquot of each dilution was spotted onto TSA plates and TSA plates containing 0.5 mM SNP. After incubation at 37 °C with 5% CO_2_ for 3–5 days, bacterial growth was observed, photographed, and recorded.

### 2.6. Fluorescent Dye Uptake Assay

Fluorescent dye uptake assays were performed as previously described [5]. Briefly, *Brucella* M5 and its derivative strains were cultured in TSB to logarithmic phase, washed twice with PBS, and adjusted to an OD_600_ of 1.0. A 200-μL aliquot of each bacterial suspension was placed into a black 96-well plate with at least three replicates per strain. Then, 2 μL of 1 mM N-phenyl-1-naphthylamine (NPN), 2 μL of 1 mM propidium iodide (PI), or 2.5 μL of 5 mM Nile red (NR) was added to each well. Fluorescence intensity of PI was measured at excitation 535 nm and emission 617 nm every 2 min for 70 min; NR fluorescence was measured at excitation 552 nm and emission 636 nm under the same time schedule; NPN fluorescence was measured at excitation 355 nm and emission 460 nm every 30 s for 600 s. To further assess outer membrane stability, bacteria were pre-incubated with polymyxin B at a final concentration of 1 mg/mL at room temperature for 1 h before the addition of NPN, and fluorescence measurements were performed as described above.

### 2.7. Cell Infection Assays

Adhesion, invasion, and intracellular survival were performed using the gentamicin protection assay as previously described [5]. Briefly, HeLa or RAW264.7 cells were seeded into 24-well plates and grown to confluent monolayers. The cells were infected with *Brucella* strains at a multiplicity of infection (MOI) of 500:1 for HeLa cells and 100:1 for RAW264.7 cells. After infection for 1 h, the cells were washed three times with PBS, and then lysed with 0.25% Triton X-100 in PBS. The lysates were serially diluted ten-fold in PBS and plated onto TSA plates to count CFUs, which represented the number of adherent bacteria. For invasion assessment, after the 1-h infection, the remaining wells were incubated with Dulbecco’s Modified Eagle Medium (DMEM) containing 100 μg/mL gentamicin for 1 h to kill extracellular bacteria, washed three times with PBS, lysed with 0.25% Triton X-100, and plated to count CFUs as the number of internalized bacteria. For intracellular survival, after the gentamicin killing step, the medium was replaced with maintenance DMEM containing 50 μg/mL gentamicin and 1% fetal bovine serum (FBS). At 1, 8, 24, and 48 h post-infection (h.p.i.), cells were lysed with 0.25% Triton X-100, and serial dilutions were plated to count CFUs. Intracellular survival curves were plotted. To investigate whether the growth defect of Δ*hisD* was due to insufficient histidine acquisition, different concentrations of histidine (final concentrations 1, 5, and 10 mM) were added to the maintenance medium during infection to evaluate whether histidine supplementation could restore the intracellular replication of the deletion mutant.

### 2.8. Indirect Immunofluorescence Assay

RAW264.7 cells were seeded into 24-well plates containing sterile coverslips and grown to confluent monolayers. Infection was performed as described above. At 24 h.p.i., the coverslips were fixed with 4% paraformaldehyde for 30 min at room temperature. After fixation, the coverslips were washed three times with PBST (0.05% Tween-20 in PBS) and then permeabilized with 0.5% Triton X-100 in PBS for 15 min at room temperature. After three washes with PBST, the coverslips were blocked with 5% bovine serum albumin in PBS (BSA-PBS) for 30 min at room temperature, followed by incubation with rabbit anti-*Brucella* polyclonal antibody (1:1000 dilution) for 1 h at 37 °C. After three washes with PBST, the coverslips were incubated with Alexa Fluor 488-conjugated goat anti-rabbit IgG (1:500 dilution, Thermo Fisher Scientific, Waltham, MA, USA) for 1 h at 37 °C in the dark. After three washes with PBS, the coverslips were blocked again with 5% BSA-PBS and then incubated with CoraLite 594-conjugated rat anti-mouse lysosomal-associated membrane protein 1 (LAMP-1) antibody (1:100 dilution, 1D4B, Proteintech, Wuhan, China) for 1 h at 37 °C in the dark. The coverslips were then washed three times with PBST and stained with 2 μg/mL DAPI for 2 min. After three additional washes with PBST and one wash with distilled water, the coverslips were air-dried and mounted using an anti-fade mounting medium (Beyotime, Suzhou, China). Co-localization of bacteria and LAMP-1 was examined using a Zeiss LSM880 confocal laser scanning microscope (Zeiss, Oberkochen, Germany). Images were processed with Adobe Photoshop 2023 (version 24.3; Adobe Inc., San Jose, CA, USA). For each sample, six random fields were selected to quantify the percentage of *Brucella* co-localized with LAMP-1.

### 2.9. Mouse Infection Assay

*Brucella* strains were cultured in TSB to logarithmic phase and adjusted to an OD_600_ of 1.0 (approximately 5 × 10^9^ CFU/mL). Forty female Balb/c mice (6–8 weeks old) were randomly divided into four groups of ten mice each. Each mouse received an intraperitoneal injection of 0.1 mL of bacterial suspension containing 1 × 10^6^ CFU. The control group received 0.1 mL of PBS. At 7 and 14 days post-infection (d.p.i.), five mice from each group were euthanized by cervical dislocation. The spleens and livers were aseptically harvested, weighed, and homogenized in 3 mL of 0.25% Triton X-100 in PBS. The homogenates were serially diluted ten-fold in PBS, spread onto TSA plates, and incubated at 37 °C with 5% CO_2_ for 3–5 days. CFUs were counted to determine the bacterial colonization loads in the spleens and livers of mice infected with the parental, deletion, and complemented strains.

### 2.10. Histological Analysis

Liver tissues were collected from infected mice at 7 and 14 d.p.i. as described above, fixed in 4% paraformaldehyde for 24 h, and then submitted to Wuhan Borf Biological Technology Co., Ltd. (Wuhan, China) for paraffin embedding and hematoxylin-eosin (H&E) staining. Whole-slide images were captured using a Pannoramic MIDI II system (3DHistech, Budapest, Hungary), and image analysis was performed with CaseViewer v2.4 (3DHistech). For each group, five randomly selected regions (13 mm^2^ per section) were examined to determine the number of granulomas per unit area. In addition, the diameters of liver granulomas were measured and statistically analyzed for three mice per group.

### 2.11. Statistical Analysis

Statistical analyses were conducted using GraphPad Prism version 9.5 (GraphPad Software, San Diego, CA, USA). Comparisons among multiple groups were assessed by one-way or two-way analysis of variance (ANOVA) followed by Dunnett’s or Tukey’s multiple comparison test. A *p*-value of less than 0.05 was considered statistically significant.

## 3. Results

### 3.1. The hisD Deletion and Complemented Strains Were Successfully Constructed

**Figure 1:**
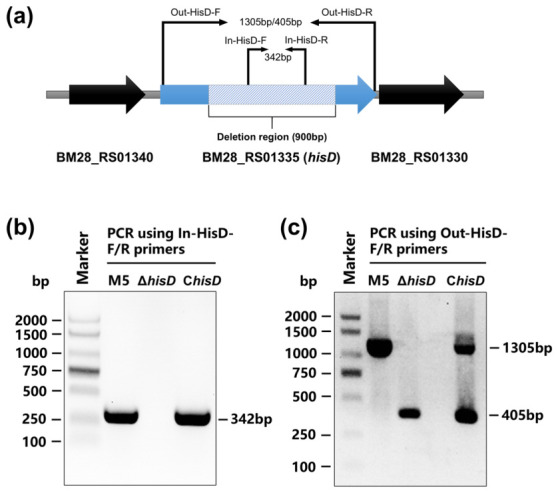
Construction and verification of the *hisD* deletion and complemented strains. (**a**) Schematic representation of the *hisD* genomic locus and the binding sites of the primers used. (**b**) PCR verification of the deletion mutant (Δ*hisD*) and complemented strain (C*hisD*) using the internal primer pair In-HisD-F/R. (**c**) PCR verification of the deletion mutant (Δ*hisD*) and complemented strain (C*hisD*) using the external primer pair Out-HisD-F/R.

The *Brucella hisD* gene is 1293 bp in length, encoding a 430-amino-acid protein that catalyzes the dehydrogenation of L-histidinol to L-histidine, serving as a key enzyme in the bacterial histidine biosynthesis pathway. Using *B. melitensis* M5 as the parental strain, we constructed the *hisD* deletion mutant Δ*hisD*, in which a 900-bp fragment was removed, accounting for 69.6% of the entire ORF (Figure 1a). Subsequently, using a miniTn7 transposon system, the *hisD* gene fragment including its predicted promoter and terminator regions was inserted into the intergenic region between *glmS* and *recG* genes on chromosome II of *Brucella* in the Δ*hisD* background, generating the complemented strain C*hisD*. To verify the deletion and complemented strains, specific primer pairs were used for PCR identification. Using the internal primers In-HisD-F/R, the parental strain M5 and the complemented strain C*hisD* both amplified a 342-bp product, whereas no amplicon was obtained from the Δ*hisD* mutant, consistent with expectations (Figure 1a,b). Using the external primers Out-HisD-F/R, the parental strain M5 produced a 1305-bp fragment; the deletion mutant, due to the 900-bp deletion, yielded a shortened 405-bp fragment; the complemented strain simultaneously generated a 405-bp small fragment (residual from the deleted endogenous locus) and a 1305-bp large fragment derived from the intact *hisD* gene carried by the miniTn7 transposon (Figure 1a,c). These results demonstrate the successful construction of the *Brucella hisD* deletion and complemented strains.

### 3.2. Deletion of hisD Blocks Histidine Biosynthesis in Brucella

**Figure 2:**
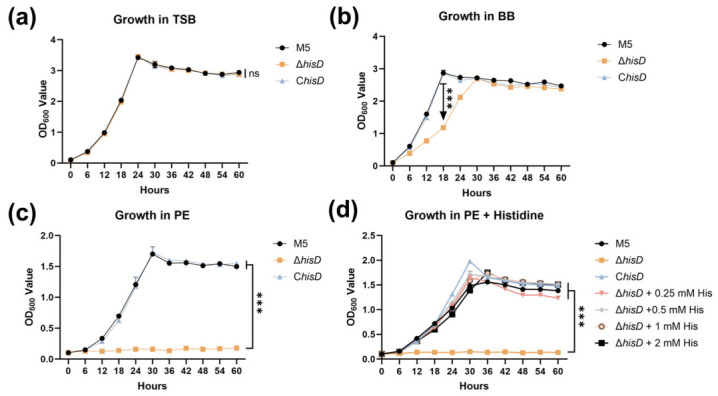
Growth curves of *Brucella* M5 and its derivative strains. (**a**) Growth in tryptic soy broth (TSB). (**b**) Growth in brucella broth (BB). (**c**) Growth in chemically defined PE medium (Plommet’s medium with 2 g/L erythritol). (**d**) Growth in PE medium supplemented with different concentrations of histidine (0.25, 0.5, 1.0, and 2.0 mM). Statistical significance was assessed using two-way ANOVA with Dunnett’s multiple comparison test (compared with the ΔhisD strain). ***, *p* < 0.001; ns, not significant.

To determine whether *hisD* deletion disrupts the histidine biosynthesis pathway as expected, we assessed the effect of *hisD* on *Brucella* growth in vitro using different culture media. In nutrient-rich TSB, the Δ*hisD* strain exhibited a growth trend similar to that of the parental strain M5 and the complemented strain C*hisD*, with no significant differences among the three (Figure 2a). In nutrient-rich BB, however, the Δ*hisD* strain grew significantly more poorly during the logarithmic phase compared with the parental and complemented strains, and its entry into the stationary phase was delayed (Figure 2b). We further evaluated the growth ability of Δ*hisD* in chemically defined PE medium. The results showed that Δ*hisD* completely failed to grow in PE medium, in contrast to the parental and complemented strains (Figure 2c). To test whether the growth defect of Δ*hisD* was due to insufficient histidine synthesis, different concentrations of histidine were added to PE medium. Supplementation with histidine significantly restored the growth of Δ*hisD* (Figure 2d). Collectively, these results indicate that *hisD* deletion interrupts the histidine biosynthesis pathway in *Brucella*, leading to insufficient histidine acquisition and consequently impairing bacterial growth in vitro.

### 3.3. Histidine Biosynthesis Deficiency Reduces the Ability of Brucella to Resist Killing by Polymyxin B and SDS

**Figure 3:**
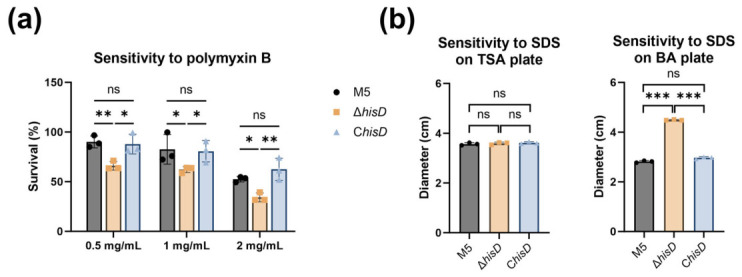
Sensitivity of *Brucella* strains to polymyxin B and sodium dodecyl sulfate (SDS). (**a**) Survival of the indicated strains after treatment with different concentrations of polymyxin B (0.5, 1.0, and 2.0 mg/mL). (**b**) Diameters of inhibition zones induced by SDS on tryptic soy agar (TSA) and brucella agar (BA). Statistical significance was determined using one-way ANOVA for (**a**) and two-way ANOVA for (**b**), followed by Tukey’s multiple comparison test. *, *p* < 0.05; **, *p* < 0.01; ***, *p* < 0.001; ns, not significant.

To explore whether *hisD* deletion affects virulence-related tolerance phenotypes of *Brucella*, we first evaluated bacterial resistance to oxidative and nitrosative stress using hydrogen peroxide and SNP (an NO donor), respectively. The Δ*hisD* strain showed no significant difference in survival ability under hydrogen peroxide or SNP treatment compared with the parental and complemented strains, indicating that *hisD* deletion does not affect the resistance of *Brucella* to oxidative or nitrosative stress (Figure S2a,b). We then assessed acid tolerance using acidic peptone water. The survival of Δ*hisD* at pH 6.5, 5.5, and 4.5 was comparable to that of the parental and complemented strains, with no significant differences (Figure S2c), suggesting that *hisD* deletion does not compromise the acid stress resistance of *Brucella*. Next, we evaluated the ability of bacteria to resist killing by the cationic antimicrobial peptide polymyxin B. Under treatment with different concentrations of polymyxin B (0.5, 1.0, and 2.0 mg/mL), the survival of Δ*hisD* was significantly lower than that of the parental and complemented strains, suggesting that *hisD* deletion may compromise the cell membrane stability of *Brucella* (Figure 3a). To further test this hypothesis, we assessed the tolerance of *Brucella* to SDS using a disk diffusion assay. On TSA plates, the Δ*hisD* strain showed no difference in SDS tolerance compared with the parental and complemented strains; however, on BA plates, Δ*hisD* exhibited significantly higher sensitivity to SDS than the parental and complemented strains (Figure 3b). Taken together with the growth defect of Δ*hisD* in BB medium, these results further suggest that histidine biosynthesis deficiency affects the membrane stability of *Brucella*.

### 3.4. Histidine Biosynthesis Deficiency Impairs Outer Membrane Stability of Brucella Under Cationic Antimicrobial Peptide Challenge

**Figure 4:**
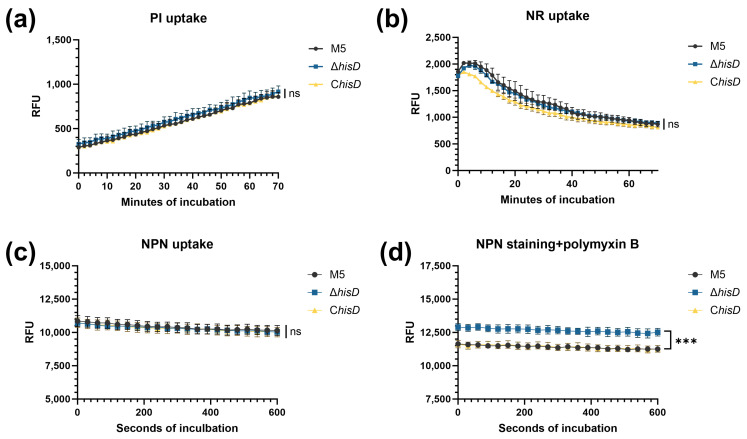
Fluorescent dye uptake by *Brucella* M5 and its derivative strains. (**a**) Propidium iodide (PI) uptake. (**b**) Nile red (NR) uptake. (**c**) N-phenyl-1-naphthylamine (NPN) uptake. (**d**) NPN uptake after pretreatment with 1 mg/mL polymyxin B for 1 h. Statistical significance was determined using two-way ANOVA followed by Dunnett’s multiple comparison test (compared with the Δ*hisD* strain). ***, *p* < 0.001; ns, not significant.

To further investigate the impact of *hisD* deletion on *Brucella* membrane stability, we used the fluorescent dyes PI, NR, and NPN to assess bacterial lipid synthesis and membrane permeability. PI, a non-membrane-permeant dye, was used to evaluate the integrity of the bacterial membrane structure. The relative fluorescence values of the parental, deletion, and complemented strains after PI staining showed no significant differences, indicating that *hisD* deletion does not affect the membrane permeability of *Brucella* (Figure 4a). NR, a lipid dye, was used to assess bacterial lipid synthesis. The relative fluorescence values after NR staining were also comparable among the three strains, suggesting that *hisD* deletion does not alter total lipid synthesis in *Brucella* (Figure 4b). We then used the membrane-hydrophobic dye NPN to evaluate outer membrane integrity. The relative fluorescence values of the three strains remained similar, indicating that *hisD* deletion does not affect the basal outer membrane structure of *Brucella* (Figure 4c). Given that *hisD* deletion increased the susceptibility of *Brucella* to polymyxin B, we performed NPN staining in the presence of polymyxin B. Under polymyxin B treatment, the NPN fluorescence of Δ*hisD* was significantly elevated and markedly higher than that of the parental and complemented strains, indicating that *hisD* deletion compromises outer membrane integrity of *Brucella* under cationic antimicrobial peptide challenge (Figure 4d). Taken together, these results demonstrate that histidine biosynthesis deficiency does not affect basal membrane integrity but attenuates outer membrane stability of *Brucella* in the presence of cationic antimicrobial peptides.

### 3.5. Histidine Biosynthesis Deficiency Impairs Intracellular Survival of Brucella Without Affecting Its Intracellular Trafficking

**Figure 5:**
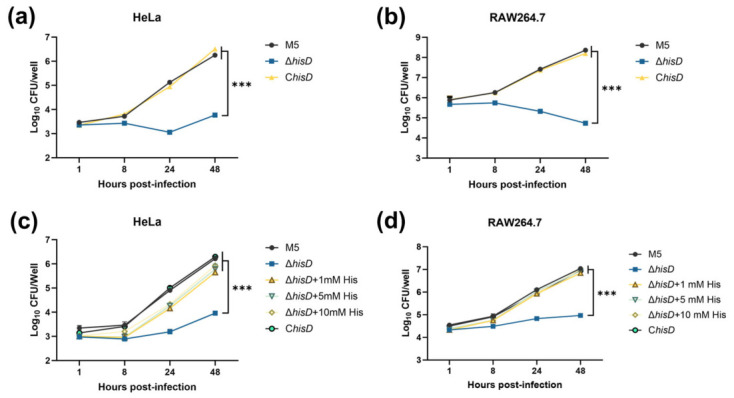
Intracellular survival of *Brucella* M5 and its derivative strains in host cells. (**a**) Survival in HeLa cells. (**b**) Survival in RAW264.7 cells. (**c**) Survival in HeLa cells: M5, Δ*hisD*, and C*hisD*, with Δ*hisD*-infected cells supplemented with 1, 5, or 10 mM histidine. (**d**) Survival in RAW264.7 cells: M5, Δ*hisD*, and C*hisD*, with Δ*hisD*-infected cells supplemented with 1, 5, or 10 mM histidine. Statistical significance was determined using two-way ANOVA followed by Dunnett’s multiple comparison test (compared with the Δ*hisD* strain). ***, *p* < 0.001.

To explore the effect of *hisD* deletion on *Brucella* infection of host cells, we first evaluated the adhesion and invasion abilities of *Brucella* towards HeLa and RAW264.7 cells. The Δ*hisD* strain showed adhesion and invasion capabilities similar to those of the parental and complemented strains, with no significant differences (Figure S3). We then assessed the intracellular survival ability of *Brucella* in these two cell types. Compared with the parental and complemented strains, Δ*hisD* barely multiplied within HeLa and RAW264.7 cells, indicating that *hisD* deletion severely impairs the intracellular replication of *Brucella* (Figure 5a,b). To test whether this phenotype resulted from insufficient intracellular histidine acquisition, we supplemented the culture medium with different concentrations of histidine during infection. Histidine supplementation significantly restored the intracellular survival of Δ*hisD* (Figure 5c,d). These data indicate that *hisD* deletion leads to insufficient histidine acquisition within host cells, thereby compromising intracellular replication of *Brucella*.

**Figure 6:**
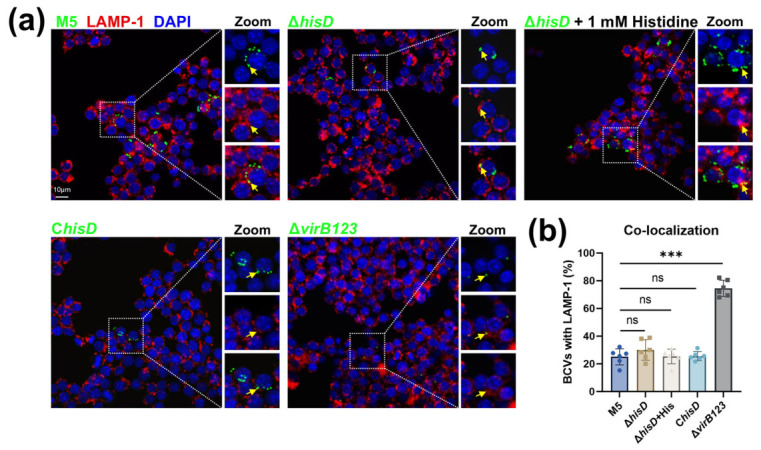
Colocalization of *Brucella* strains with the lysosomal marker LAMP-1 in RAW264.7 cells. (**a**) Representative immunofluorescence images showing colocalization of *Brucella*-containing vacuoles (BCVs) with LAMP-1 at 24 h post-infection. Yellow arrows indicate BCVs that colocalize with LAMP-1. (**b**) Quantitative analysis of the percentage of BCVs positive for LAMP-1 colocalization. Statistical significance was determined using one-way ANOVA followed by Dunnett’s multiple comparison test (compared with the parental strain M5). ***, *p* < 0.001; ns, not significant.

Intracellular survival of *Brucella* also depends on its ability to escape lysosomal degradation [19]. Using an indirect immunofluorescence assay, we analyzed the co-localization of *Brucella* with the lysosomal marker LAMP-1. As shown in Figure 6a, the number of intracellular replicating bacteria of Δ*hisD* was significantly reduced compared with the parental strain M5 and the complemented strain C*hisD*. When 1 mM histidine was added to the cell culture medium, the intracellular replication level of Δ*hisD* was markedly increased, which is consistent with the intracellular survival curves determined by the gentamicin protection assay. Fluorescence co-localization analysis revealed that, despite the reduced intracellular replication, the majority of Δ*hisD* bacteria still did not co-localize with LAMP-1 (Figure 6a). Quantitative analysis of the percentage of intracellular *Brucella* co-localized with LAMP-1 showed that at 24 h.p.i. of RAW264.7 cells, the co-localization percentage of Δ*hisD* was approximately 30%, which was not significantly different from that of the parental strain, the complemented strain, or the histidine-supplemented deletion strain. In contrast, the positive control strain Δ*virB123* exhibited a co-localization efficiency of approximately 75%, which was significantly higher than that of all other groups (Figure 6b). These results indicate that the impaired intracellular survival caused by *hisD* deletion is not associated with defects in the ability of *Brucella* to escape lysosomal degradation during intracellular trafficking.

### 3.6. Histidine Biosynthesis Deficiency Attenuates the Pathogenicity of Brucella in Mice

**Figure 7:**
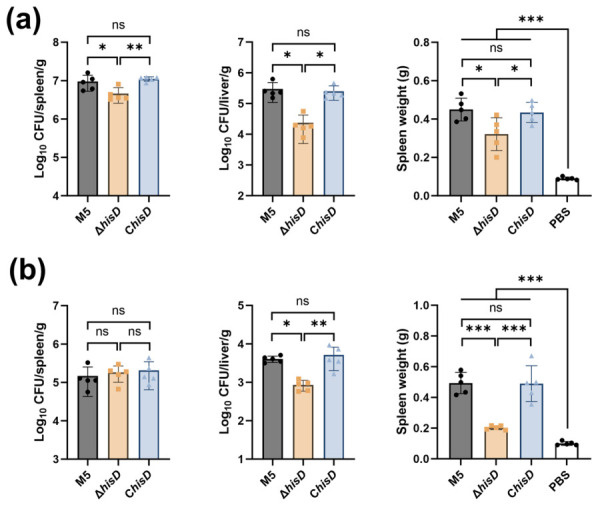
Virulence of *Brucella* strains in a mouse model. (**a**) Bacterial loads in the spleen and liver, and spleen weight, at 2 weeks post-infection (w.p.i.) with 1 × 10^6^ CFU. (**b**) Bacterial loads in the spleen and liver, and spleen weight, at 4 w.p.i. with 1 × 10^6^ CFU. Statistical significance was determined using one-way ANOVA followed by Tukey’s multiple comparison test. *, *p* < 0.05; **, *p* < 0.01; ***, *p* < 0.001; ns, not significant.

To determine whether the histidine biosynthesis defect resulting from *hisD* deletion affects the virulence of *Brucella*, we evaluated the pathogenicity of *Brucella* using a mouse infection model. We first assessed bacterial loads in the spleens and livers of mice, as well as the degree of splenomegaly, at 7 and 14 d.p.i. with the parental, deletion, and complemented strains. At 7 d.p.i., the bacterial loads of Δ*hisD* in both the spleen and liver were significantly lower than those of the parental and complemented strains, and the spleen weight of mice infected with Δ*hisD* was also significantly lower than that of mice infected with the other two strains (Figure 7a). At 14 d.p.i., the bacterial loads of Δ*hisD* in the spleen were not significantly different from those of the parental and complemented strains, but the bacterial loads in the liver remained significantly lower. Moreover, the splenomegaly induced by Δ*hisD* infection was consistently less severe than that induced by the parental and complemented strains (Figure 7b). These results indicate that *hisD* deletion reduces the ability of *Brucella* to induce splenomegaly, as well as its colonization ability in the liver and in the spleen during the early stage of infection.

**Figure 8:**
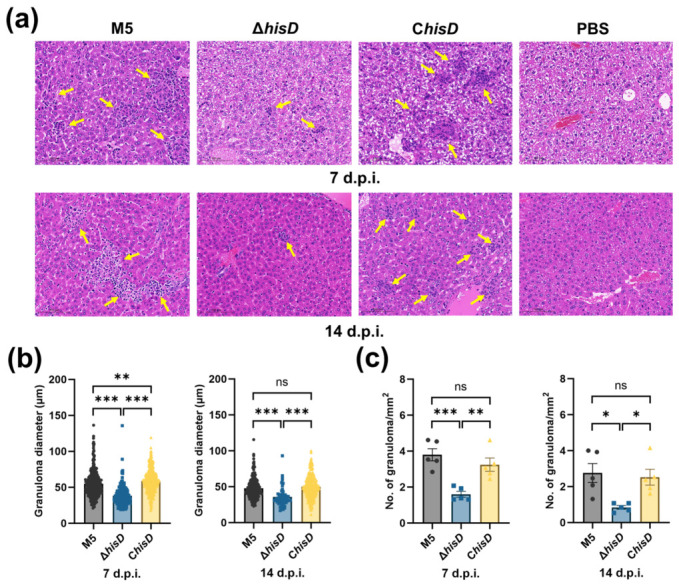
Histopathological analysis of liver sections from mice infected with *Brucella* strains. (**a**) Representative H&E-stained liver tissues at 7 and 14 d.p.i. (400× magnification). Yellow arrows indicate granulomas. (**b**) Diameter of granulomas. (**c**) Number of granulomas per mm^2^. Statistical significance was determined using one-way ANOVA followed by Tukey’s multiple comparison test. *, *p* < 0.05; **, *p* < 0.01; ***, *p* < 0.001; ns, not significant.

To further confirm that *hisD* deletion reduces *Brucella* virulence, we examined the histopathological changes in the livers of infected mice. At 7 and 14 d.p.i., histopathological sections showed that the ability of Δ*hisD* to induce granuloma formation in the liver was significantly lower than that of the parental and complemented strains, whereas the livers of PBS-treated mice exhibited no obvious pathological changes (Figure 8a). To quantitatively analyze liver granuloma formation, we measured the diameter of granulomas and counted the number of granulomas per unit area (mm^2^). The granuloma diameter induced by Δ*hisD* at both 7 and 14 d.p.i. was significantly smaller than that induced by the parental and complemented strains (Figure 8b). Similarly, the number of granulomas per unit area was significantly lower in Δ*hisD*-infected mice compared with mice infected with the parental or complemented strain (Figure 8c). These data further demonstrate that *hisD* deletion induced histidine biosynthesis deficiency significantly attenuates *Brucella* virulence.

## 4. Discussion

In this study, we successfully constructed a *hisD* deletion mutant (Δ*hisD*) and a complemented strain (C*hisD*) based on the *B. melitensis* strain M5, and systematically evaluated the impact of histidine biosynthesis deficiency on *Brucella* virulence-related phenotypes. Our results demonstrated that *hisD* deletion rendered *Brucella* completely dependent on exogenous histidine for growth in chemically defined medium, confirming the essential role of HisD in *de novo* histidine biosynthesis in *Brucella*. Interestingly, the growth defect of Δ*hisD* was observed in BB but not in TSB, despite both being nutrient-rich media. A tentative explanation for this difference may lie in the distinct free histidine concentrations or amino acid compositions between the two media. TSB contains soybean digest, which is rich in free amino acids including histidine, whereas BB relies on animal tissue digest and yeast extract, which may provide lower levels of free histidine. Consequently, in TSB, the exogenous histidine might be sufficient to support the growth of Δ*hisD*, while in BB, the histidine supply falls below the threshold required for normal growth. More importantly, we report for the first time that histidine biosynthesis deficiency compromises the outer membrane stability of *Brucella* in a condition-dependent manner: under normal culture conditions, the outer membrane integrity of Δ*hisD* was not markedly affected; however, in the presence of the cationic antimicrobial peptide polymyxin B, its outer membrane integrity was significantly impaired. Furthermore, *hisD* deletion severely impaired the intracellular replication of *Brucella* in macrophages and HeLa cells, and significantly reduced bacterial colonization in the liver and spleen as well as overall pathogenicity in a mouse model. These findings systematically reveal the multifaceted effects of histidine biosynthesis deficiency on *Brucella* virulence and provide a theoretical basis for developing anti-*Brucella* strategies targeting the histidine biosynthesis pathway.

HisD is a key enzyme in bacterial histidine biosynthesis, catalyzing the last two steps of the pathway [20]. Our in vitro growth assays showed that *hisD* deletion completely abolished the histidine biosynthetic pathway in *B. melitensis*, consistent with a previous report that *hisD* deletion affects the growth of *B. abortus* [21]. Intracellular survival assays revealed that *hisD* deletion significantly impaired the intracellular replication of *B. melitensis*, which aligns with findings from transposon mutant library screens identifying *hisD* as a gene essential for intracellular survival of *B. abortus* and *B. suis* [12,13]. These observations suggest that different *Brucella* species require *de novo* histidine biosynthesis to obtain sufficient histidine during intracellular replication. Indirect immunofluorescence analysis indicated that the reduced intracellular survival caused by histidine biosynthesis deficiency was not associated with impaired escape from lysosomal fusion, further suggesting that this intracellular survival defect results from the inability of *Brucella* to acquire adequate exogenous histidine within the host intracellular niches. Interestingly, Dwivedy et al. reported that the *de novo* histidine biosynthesis pathway in *Mycobacterium tuberculosis* protects the bacterium against host interferon-gamma (IFN-γ)-mediated histidine starvation [22]. Given that IFN-γ also plays a critical role in defense against *Brucella* infection [23,24], it is highly plausible that IFN-γ limits histidine acquisition by *Brucella* through a similar mechanism, thereby attenuating its virulence.

Histidine is not only an essential nutrient for bacterial growth but also a determinant of outer membrane phenotypes when its supply is limited. In this study, we observed that in the presence of polymyxin B, *hisD* deletion significantly enhanced the uptake of the outer membrane-lipophilic dye NPN by *Brucella*, whereas no such phenomenon was observed under normal non-stressed conditions. Together with the reduced resistance of Δ*hisD* to polymyxin B and SDS, these results strongly suggest that histidine auxotrophy compromises the outer membrane stability of *Brucella* under bactericidal stress. To the best of our knowledge, reports on this phenotype are rare. Notably, overexpression of the histidine biosynthesis gene cluster in *Salmonella* Typhimurium significantly affects bacterial outer membrane morphology, inducing the formation of long filaments and large balloon-like structures [25]. In escape mutants obtained by mutagenesis, numerous mutations occurred in cell envelope-related genes, accompanied by altered bacterial morphology, increased susceptibility to bactericidal agents, and enhanced autolytic activity [25], suggesting a link between histidine biosynthesis and bacterial outer membrane morphology. The observation that *hisB* deletion in *B. abortus* leads to long-chain bacterial growth further supports an association between histidine biosynthesis and bacterial morphology [26]. Additionally, it has been shown that histidine auxotrophic mutants of *Brucella* (e.g., *hisA*, *hisB*, *hisC*, and *hisD* mutants) are highly sensitive to copper ions, and this sensitivity depends on the expression of two periplasmic proteins, OppA1 and OppA2, of the oligopeptide transport system (*opp* operon) [21]. Interestingly, another oligopeptide ABC transporter, YejABEF, has been reported to be required for resistance of *B. melitensis* to polymyxin B killing [27]. Therefore, whether the expression of OppA induced by histidine auxotrophy also contributes to the tolerance of *Brucella* to polymyxin B and SDS warrants further investigation.

Beyond affecting nutrient acquisition and outer membrane stability, histidine biosynthesis deficiency may indirectly interfere with other virulence-related metabolic pathways. The histidine biosynthesis pathway shares the precursor molecule PRPP with the *de novo* nucleotide biosynthesis pathway, and the histidine biosynthesis by-product 5-aminoimidazole-4-carboxamide ribonucleotide (AICAR) can enter the bacterial purine nucleotide biosynthesis pathway [28,29]. The *de novo* nucleotide biosynthesis pathway is known to be essential for *Brucella* virulence, as deletion of multiple genes involved in this pathway impairs intracellular survival and replication [5,12,13]. In particular, deletion of the purine biosynthesis gene *purD* significantly affects lipid synthesis and membrane homeostasis in *Brucella*, and notably, the *purD* mutant also exhibits enhanced NPN staining [5], which is consistent with the NPN staining results obtained for Δ*hisD* in this study. The interplay between these two biosynthetic pathways likely contributes to the virulence phenotypes of *Brucella*. Furthermore, it has been reported that the histidine utilization repressor HutC senses the histidine metabolite urocanic acid (UCA) to negatively regulate histidine utilization, while simultaneously acting as a co-activator to positively regulate expression of the VirB system [30]. The VirB system encodes the T4SS, a crucial virulence determinant of *Brucella* that is essential for intracellular survival and replication [31]. Histidine biosynthesis deficiency leads to reduced histidine utilization and consequently lower levels of UCA, which may attenuate the activation of the VirB system by HutC, thereby reducing *Brucella* virulence. Together, these lines of evidence indicate that histidine metabolism plays multiple roles far beyond that of a simple nutrient in the adaptation of *Brucella* to the host intracellular environment, and the underlying molecular mechanisms merit further investigation. Of note, the last two steps of histidine biosynthesis catalyzed by HisD are completely absent in mammals, making HisD an ideal target for anti-*Brucella* drug development [32,33]. HisD inhibitors have already shown significant anti-*Brucella* activity at the macrophage level [14].

This study systematically evaluated the effects of histidine biosynthesis deficiency on *Brucella* virulence-related phenotypes, but several limitations remain. First, one methodological consideration is the use of the attenuated vaccine strain *B. melitensis* M5 as the parental strain, rather than a fully virulent reference strain such as *B. melitensis* 16M or *B. abortus* 2308. M5 is a well-characterized, smooth-type attenuated strain that retains key virulence-associated features (smooth LPS and intracellular replication capacity) [5,7], making it a relevant model for initial functional characterization of *hisD*. However, it remains formally possible that the conditional membrane sensitivity phenotypes observed here (e.g., increased susceptibility to polymyxin B and SDS) result from an interaction between the *hisD* deletion and pre-existing attenuating mutations in the M5 genome, rather than being a direct and exclusive consequence of histidine auxotrophy. Therefore, while our results strongly support an essential role for HisD in *Brucella* intracellular survival and outer membrane stability under stress, future studies using fully virulent strains (e.g., *B. melitensis* 16M or *B. abortus* 2308) are warranted to validate these findings in a wild-type genetic background. Second, the molecular mechanism by which *hisD* deletion affects outer membrane stability of *Brucella* is still unclear. How histidine deficiency specifically influences the modification or assembly of membrane components (e.g., LPS or outer membrane proteins) requires further investigation. Third, HutC serves as a molecular bridge between histidine metabolism and T4SS expression, and the regulatory changes in HutC activity in the *hisD* deletion background warrant in-depth exploration. How histidine deficiency affects the expression of *virB* and other virulence genes via HutC is a key question for understanding the attenuation of virulence caused by this metabolic defect. Fourth, although we have demonstrated that the *hisD* deletion mutant is significantly attenuated in a mouse model, its immunoprotective efficacy as a live attenuated vaccine candidate has not yet been evaluated. Previous studies have indicated that auxotrophic live vaccines hold promise for *Brucella* vaccine development [34,35]. Future studies should assess the ability of Δ*hisD* to induce protective immune responses and its immunoprotective efficacy, based on a thorough understanding of its safety profile. Finally, while HisD is widely recognized as a promising anti-*Brucella* drug target, the phenotypic evidence obtained from a gene deletion perspective in this study provides functional genomic support for the validity of this target. The in vivo efficacy of HisD inhibitors should be further evaluated in animal infection models.

Beyond targeting single metabolic enzymes like HisD, the global rise of antimicrobial resistance (AMR) calls for diversified strategies to combat brucellosis and other bacterial infections [16]. Several innovative approaches have emerged to overcome resistance mechanisms, particularly those associated with the Gram-negative outer membrane and efflux pumps. The “Trojan horse” strategy, which uses siderophores to deliver antibiotics into bacteria, has shown potential against intracellular pathogens [36]. Targeting bacterial metallophores to disrupt metal homeostasis can attenuate pathogenicity without direct killing pressure [37]. Bacteriophage therapy offers another avenue by specifically lysing drug-resistant strains [38]. Integrating such alternative antimicrobial paradigms with metabolic-targeted inhibitors like those against HisD could provide a synergistic defense against AMR in brucellosis, an area warranting future investigation.

## 5. Conclusions

In this study, the *hisD* deletion mutant Δ*hisD* and the complemented strain C*hisD* of *B. melitensis* were successfully constructed. *hisD* deletion disrupted *de novo* histidine biosynthesis, making the mutant strictly dependent on exogenous histidine for growth. Histidine biosynthesis deficiency compromised outer membrane stability in a condition-dependent manner and severely impaired intracellular replication in HeLa and RAW264.7 cells. It also reduced bacterial colonization in mouse liver and spleen, attenuated granuloma formation, and diminished overall virulence. These findings demonstrate that HisD is essential for *Brucella* intracellular survival and virulence. Given the absence of histidine biosynthesis in mammals, HisD represents a promising target for anti-*Brucella* drug development, and Δ*hisD* is a potential live attenuated vaccine candidate.

## Figures and Tables

**Table 1:** Bacterial strains and plasmids used in this study.

Strains and Plasmids	Description	Sources
Strains		
*B*. *melitensis* M5	Attenuated strain; smooth phenotype	CVCC
Δ*hisD*	The *hisD* deletion mutant deprived from M5	This study
C*hisD*	The *hisD* complemented strain	This study
Δ*virB123*	The *virB1*, *virB2* and *virB3* deletion mutant	[4]
*E. coli* DH5α	F−, φ80d*lacZ* ΔM15, Δ*(lacZYA-argF)*U169, *recA1*, *endA1*, *hsdR17(rk−, mk+)*, *phoA*, *supE44*, *thi-1*, *gyrA96*, *relA1*, λ−	TIANGEN
Plasmids		
pKB	Kan^R^; SacB	[4]
pMiniTn7TK	Amp^R^, Kan^R^; miniTn7 transposon	[4]
pHelp1	Amp^R^; Tn7 transposase TnsABCD	[4]
pKB-Δ*hisD*	pKB plasmid carrying the upstream and downstream homologous fragments of the *hisD* gene	This study
pMiniTn7TK-C*hisD*	pMiniTn7TK carrying the *hisD* gene with its promoter and terminator regions	This study

**Table 2:** Primers used in this study.

Primers	Sequence (5′-3′)	Source
HisD-UF	GGTACCCGGGGATCCTCATTGGCTGCATCATCGTG	This study
HisD-UR	ATAATCGCCGATCACGTCGAAGCGGCGTGAATAAT	This study
HisD-DF	TCACGCCGCTTCGACGTGATCGGCGATTATGTGGG	This study
HisD-DR	TGCCTGCAGGTCGACAGCACAGCATCGACAAAAGG	This study
CHisD-F	CATGAGCTCACTAGTGGATCCCGATCTCCAGTATCTCGCCA	This study
CHisD-R	GCAAGGCCTTCGCGAGGTACCCAGCGAGAGAATATGCGTGG	This study
In-HisD-F	CCGATGGCAATCTCAATCCG	This study
In-HisD-R	CAAAGGCCTCGTCATTGGTC	This study
Out-HisD-F	GTCACAACGCTCAGACAGAC	This study
Out-HisD-R	GGCAGTCATGGTCCCTCATA	This study

## Data Availability

The original contributions presented in this study are included in the article/Supplementary Material. Further inquiries can be directed to the corresponding authors.

## Supplementary Materials

The following supporting information can be downloaded at: https://www.mdpi.com/article/10.3390/microorganisms14061323/s1, Figure S1 Histidine biosynthetic pathway in *Brucella melitensis*; Figure S2 Sensitivity of *Brucella* strains to stress factors; Figure S3 Adhesion and invasion of *Brucella* strains to host cells.

## Abbreviations

The following abbreviations are used in this manuscript:

HisD	Histidinol dehydrogenase
PRPP	5-phosphoribosyl-1-pyrophosphate
TSA	Tryptic soy broth
BB	Brucella broth
CFU	Colony-forming units
SDS	Sodium dodecyl sulfate
PI	Propidium iodide
NPN	N-phenyl-1-naphthylamine
NR	Nile red
LAMP-1	Lysosomal-associated membrane protein 1
UCA	Urocanic acid
T4SS	Type IV secretion system
LPS	Lipopolysaccharide
