# Supplementary material for: Disruption of Histidine Biosynthesis Impairs Outer Membrane Stability and Intracellular Survival of Brucella melitensis, Resulting in Attenuated Virulence
Source: Microorganisms. 2026 Jun 12;14(6):1323. doi: 10.3390/microorganisms14061323 (PMC13303184; doi:10.3390/microorganisms14061323)
Supplement: Supplementary file 1 [file microorganisms-14-01323-s001.zip › microorganisms-4362250-supplementary.pdf]

Supplementary Materials

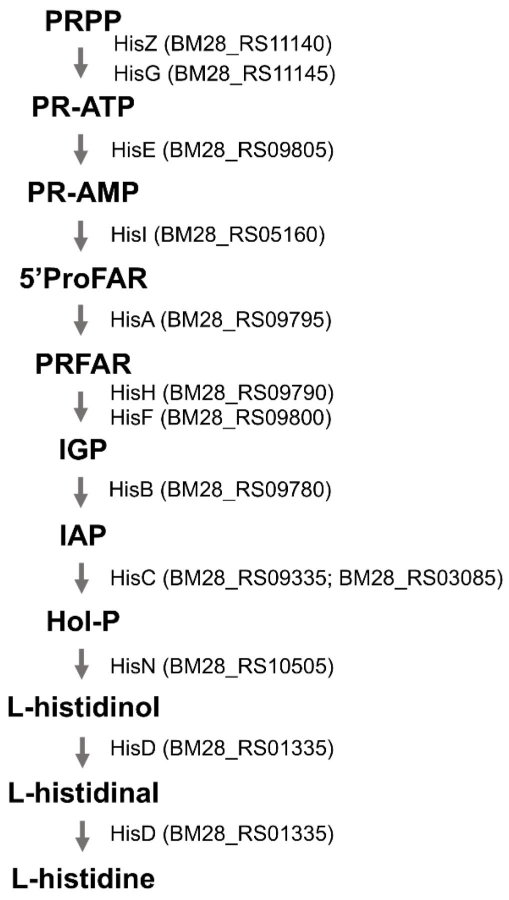

Figure S1. Histidine biosynthetic pathway in *Brucella melitensis*. The pathway was adapted from the KEGG metabolic map for *B. melitensis* strain M28 (https://www.kegg.jp/kegg-bin/show_pathway?bmz00340). Abbreviations: PRPP, 5-phospho-α-D-ribose 1-diphosphate; PR-ATP, N1-(5-phosphoribosyl)-adenosine triphosphate; PR-AMP, N1-(5-phosphoribosyl)-adenosine monophosphate; 5'ProFAR, N1-(5-phosphoribosyl)-formimino-5-aminoimidazole-4-carboxamide ribonucleotide; PRFAR, N1-[(5-phosphoribulosyl)formimino]-5-aminoimidazole-4-carboxamide ribonucleotide; IGP, imidazole-glycerol phosphate; IAP, imidazole acetol-phosphate; Hol-P, L-histidinol phosphate; HisZ, ATP phosphoribosyltransferase regulatory subunit; HisG, ATP phosphoribosyltransferase; HisE, phosphoribosyl-ATP pyrophosphohydrolase; HisI, phosphoribosyl-AMP cyclohydrolase; HisA, phosphoribosylformimino-5-aminoimidazole-4-carboxamide ribonucleotide isomerase; HisH, imidazole glycerol phosphate synthase subunit HisH; HisF, imidazole glycerol phosphate synthase subunit HisF; HisB, imidazoleglycerol-phosphate dehydratase; HisC, histidinol-phosphate aminotransferase; HisN, histidinol-phosphatase; HisD, histidinol dehydrogenase.

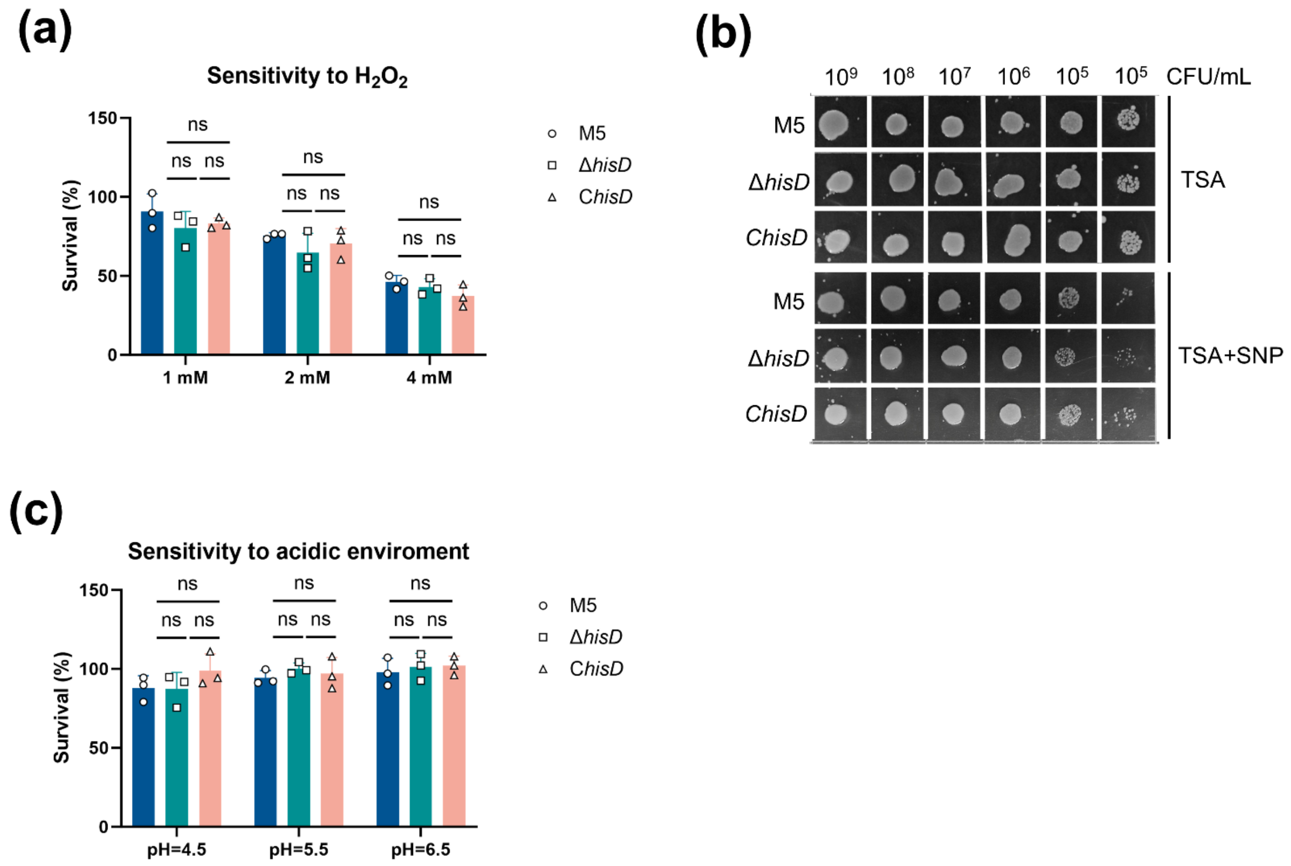

Figure S2. Sensitivity of *Brucella* strains to stress factors. (**a**) Survival after H_2O_2 treatment (1, 2, and 4 mM). (**b**) Survival after incubation in acidic peptone water (pH 4.5, 5.5, and 6.5). (c) Growth on TSA plates with or without 0.5 mM SNP. Statistical significance was determined using one-way ANOVA followed by Tukey's multiple comparison test. ns, not significant.

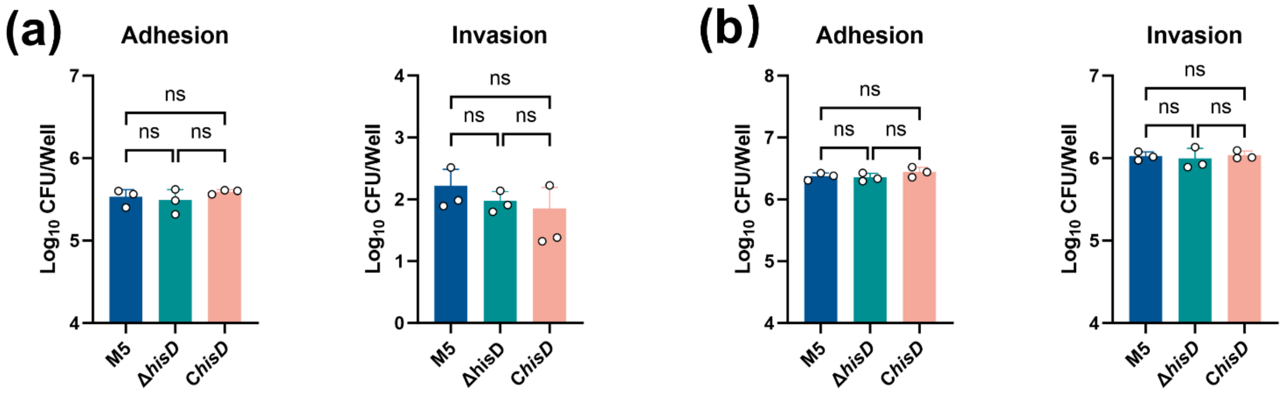

Figure S3. Adhesion and invasion of *Brucella* strains to host cells. (**a**) Adhesion and invasion to HeLa cells. (**b**) Adhesion and invasion to RAW264.7 cells. Statistical significance was determined using one-way ANOVA followed by Tukey's multiple comparison test. ns, not significant.
